# The Higher Professional Duties of Dentists

**Published:** 1882-02

**Authors:** 


The Higher Professional Duties of Dentists.—In a recent number of the discontinued *Dental Miscellany* appears an editorial note on "Dentists' Duties," in which the keynote is struck of one highly important stage of professional progress.

It is a duty there but vaguely hinted at, which we have many times argued in the affirmative with our fellow practitioners, and urged with great stress upon those who are just entering upon the practice of the specialty. It is this: the necessity for educating the patients to a higher appreciation of the dentist than as a mere machinist.

This leads to the question, what is the first essential to one who would make this innovation in behalf of a higher appreciation by the people of the true professional character of the services rendered by the educated, skilled and conscientious dental surgeon?

We will answer briefly. He must present himself as one capable of something higher than a rubber plate maker, tooth puller, or a tooth filler, either as a "new departurist," or as a contourist.

Neither one of these, nor all combined, practiced never so faithfully and conscientiously, can leave other impression upon the patients mind than that dentistry is mechanical and that the dentist is to be held to account for his work just as the plumber or gas fitter.

We regret to say that the great majority of medical men are as little conversant with the aim, object and results of intelligent dental practice, as are the patients, as is evidenced by the frequent cases of blind treatment of facial neuralgia, antral disease, alveolar necrosis, Rigg's disease, and other diseases not so intimately connected with the oral cavity. These cases fall into our hands more frequently by accident than by intention of the medical adviser, and why does he not seek advice or assistance from the dentist? Because not more than one in a hundred of them know that dentistry is not confined to the strictly mechanical treatment of the teeth.

One of the objects of this journal is to bring before the eyes of the medical profession, the full scope of the work of the educated, progressive D. D. S., to bridge the chasm, which now separates us, and thus to establish a sympathy, based upon actual knowledge of dental surgeons' work, which shall make medical men co-workers, instead of depreciators and antagonists.

The good to be accomplished will be reciprocal, for, as expressed in a letter to us by an eminent dental surgeon and educator; "*The peculiar need, both of the medical and dental professions, is just such a journal. The former needs to know about dental matters from the special standpoint. The latter requires that broadening of views which can come only out of an introduction into the field of general medicine.*"

And this opinion has been reiterated by many of our correspondents from amongst the leading lights of our specialty.

